# Achieving agricultural sustainability through soybean production in Iran: Potential and challenges

**DOI:** 10.1016/j.heliyon.2024.e26389

**Published:** 2024-02-16

**Authors:** Parastoo Majidian, Hamid Reza Ghorbani, Mostafa Farajpour

**Affiliations:** Crop and Horticultural Science Research Department, Mazandaran Agricultural and Natural Resources Research and Education Center, Agricultural Research, Education and Extension Organization (AREEO), Sari, Iran

**Keywords:** Diversity, Environmental impacts, Productivity, Supply chain

## Abstract

The utilization of soybean as a key oil crop to enhance sustainable agriculture has garnered significant attention from researchers. Its lower water requirements compared to rice, along with its reduced environmental impact, including greenhouse gas emissions, improved water quality, enhanced biodiversity, and efficient resource utilization, make it an attractive option. Unfortunately, Iran, like many other developing countries, heavily relies on soybean imports (over 90%) to meet the demand for oil and protein in human and livestock food rations. The decline in soybean production, coupled with diminishing cultivation areas, yield rates, and increasing import needs, underscores the urgent need to address the challenges faced in Iran. The decline in soybean production in the country can be attributed to various factors, including environmental stresses (both biotic and abiotic), limited variation in soybean cultivars, inadequate mechanization for cultivation, and economic policies. Hence, this review provides a comprehensive overview of the current status of soybean production in Iran and highlights its potential to enhance sustainable agriculture. Additionally, it examines the challenges and constraints associated with soybean cultivation, such as environmental changes and unbalanced marketing, and explores potential solutions and management strategies to bridge the gap between small-scale and large-scale production. Given the increasing global demand for plant-based protein and the significance of the feed industry, studying the limitations faced by countries with slower soybean production growth can shed light on the issues and present opportunities to capitalize on novel soybean advancements in the future. By addressing these challenges and unlocking the potential of soybean cultivation, Iran can contribute to sustainable agricultural practices and attain a more resilient food system.

## 1 Introduction

Soybean is an economically import oilseed crop which is mainly cultivated for its high protein (40%), oil content (20%) and other valuable nutritional materials. This crop belongs to leguminose family which has the ability of reinforcement of soil due to nitrogen biologically fixation following by productivity and stability enhancement of soil texture and reduction of chemical fertilizer usage for next crops in rotation [1]. In the world, the various consumptions of soybean are referred to food (for human and livestock), medical usages and industrial products which cause the increase need for its production. The top five superior countries with the highest rate of soybean production consists of United states, Brazil, Argentina, China and India with total production area under cultivation of 129,523,964 ha and production rate of 353,370,766 t [2]. Among them, USA has become the major soybean production region by rate of 87,896,985 t, yearly.

**Fig. 1:**
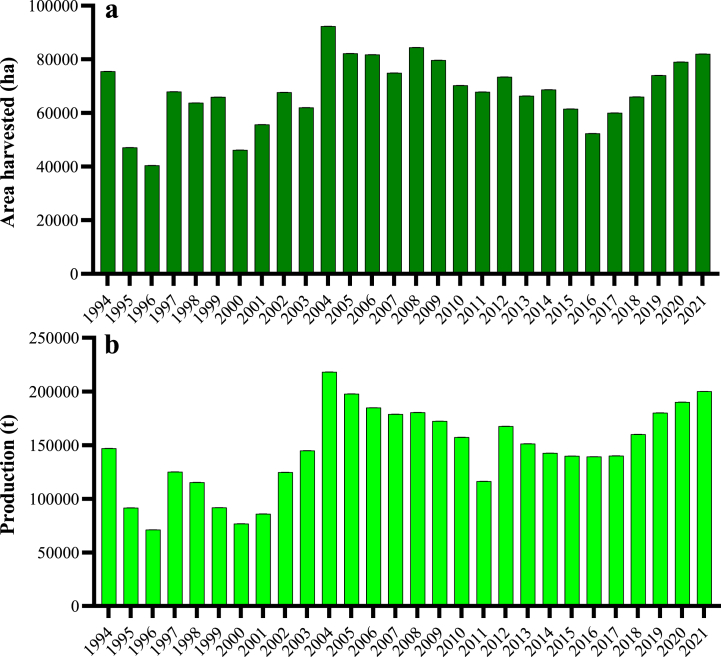
Area harvested (a) and total production (b) of soybean in Iran during 1994–2021.

**Fig. 2:**
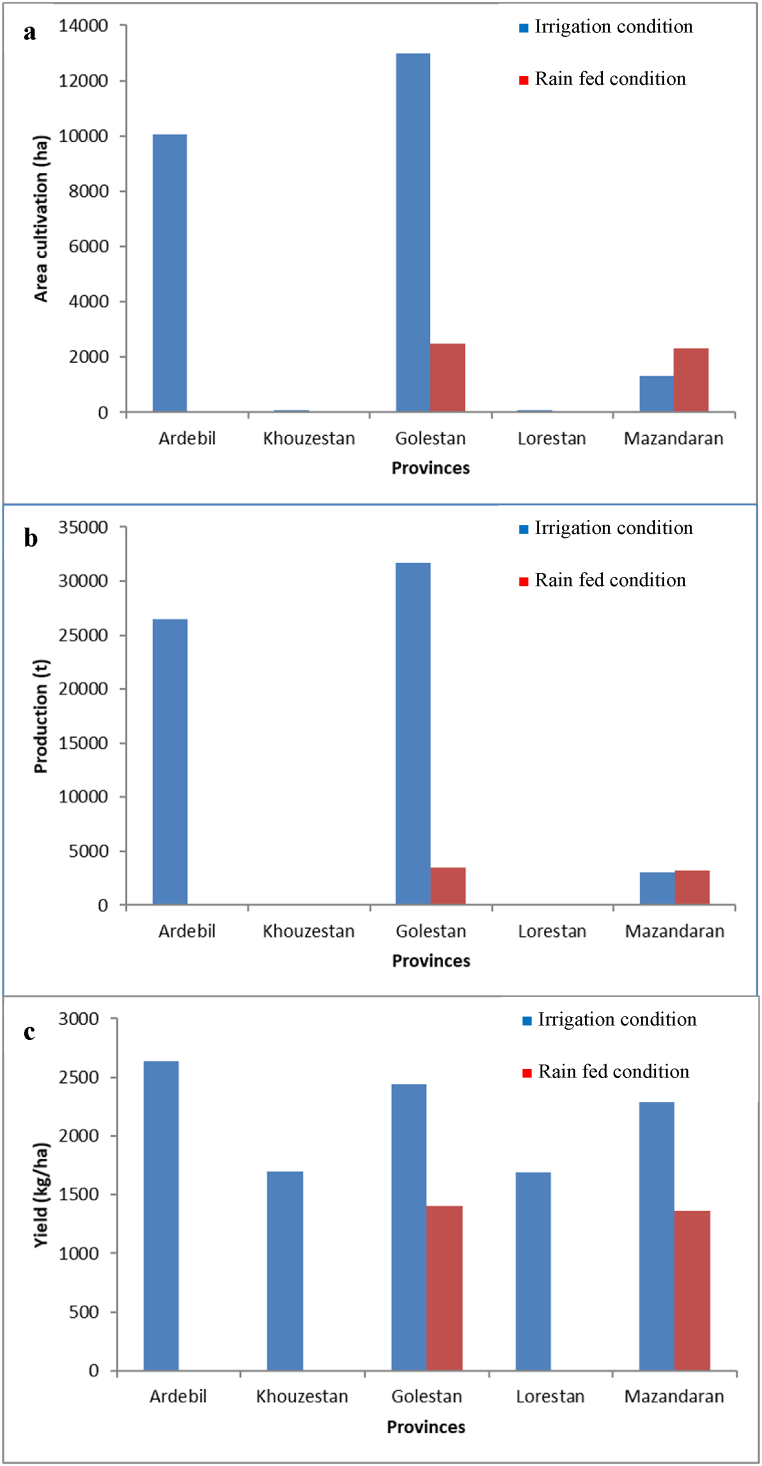
Area cultivation (a), production (b) and seed yield (c) of soybean in different provinces of Iran.

The cultivation of soybean in Iran could be traced back 90 years ago, when some soy varieties were imported to Iran from China by soybean dealers. Based on Fig. 1(a, b), the trend of soybean plantation didn't show the balanced process during 1994–2021 emphasizing the area harvested of 82,000 ha and production level of 200,000 t in 2021 (lower than recent decades) [2]. Generally, soybean cultivation is distributed in five different provinces of the country, including north (Ardabil, Golestan, and Mazandaran), west (Lorestan), and south (Khuzestan) with productivity range of about 2.5 and 1.5 t/ha under irrigated and rain-fed conditions, respectively (Fig. 2a–c). The provinces of Golestan, Ardabil, and Mazandaran are the leading producers of soybeans in Iran, with production of 35,200, 26,500, and 6251 t/ha, respectively [3].

In northern part of Iran, soybeans can be planted in two seasons, spring and summer, after wheat/corn harvest. Due to seasonal rainfalls and lack of irrigation systems, most farmers prefer to plant soybeans under rain-fed conditions in well-timed of spring (mid-May/early June). To achieve optimum yield, it is necessary to use artificial irrigation systems in summer seasons (mid-June/Early-July). While, soybean cultivation is widespread around late-June due to early warming season in southern areas of Iran [4].

In recent years, various soybean cultivars with specific traits have been bred by soybean breeders and released under the supervision of Seed and Plant Improvement Institute, as presented in Table 1. Totally, soybean varieties are classified into 13 maturity groups (MGs) ranging from MG 000 for the earliest-maturing varieties to MG X for the latest-maturing ones based on tits maturity time [5]. Considering Iranian soybean varieties and their areas under cultivation, there are three types of maturity groups, including 1) the early maturing group as MG000/MG00 suitable for temperate and cold regions of Iran as Ardabil, West Azerbaijan, and Lorestan provinces, 2) the medium maturity group (MG V) appropriate for Golestan and Mazandaran provinces, and 3) the latest maturity group of (MG VI) compatible with the region of Khuzestan province (southern part of Iran). In terms of number of days to maturity displayed in Iranian soybean cultivars, the growth period of early-maturing varieties is about 100–110 days, while the late-maturing varieties are approximately 120–150 days, depending on various cultivation areas [6].

**Table 1:** Characteristics of commercial Iranian soybean cultivars: varietal traits and descriptions.

Cultivar^a^	SD (t/ha)	GP (d)	Maturity Group	Growth type	1000-seed weight (g)	Oil content (%)	Protein content (%)	Flower color	Cultivation zone
Gorgan 3	3–3.5	150	4	Determinate	180	18	39	Purple	M & G
Williams	2.5–3	120	3	Indeterminate	150	21	37	White	A & L
Zan	2.5	115	3	Indeterminate	180	22	36	Purple	A & L
Sahar	2.5–3	135	4	Semi-determinate	140	21	37	White	M & G
Telar	3.8	140	5	Semi-determinate	160	20	37	White	M
Sari	4.1	150	5	Semi-determinate	170	22	37	Purple	M
Nekador	3.6	145	5	Semi-determinate	180	22	36	Purple	M
Katol	3.3	160	5	Semi-determinate	200–220	20	39	Purple	G
Caspian	3.5	145	5	Semi-determinate	180	23	35	White	M
Saland	2.4	125	6	Determinate	155	22	38	Purple	K
Saman	3.7	150	5	Indeterminate	155	21.8	38.4	Purple	G
Kosar	3.3	108	2	Indeterminate	135	22	37	Purple	L
Amir	3.5	160	5	Determinate	135	22	37	Purple	G
Tapor	4.9	143	5	Semi-determinate	195	22	37	White	M
Saba	3.8	118	2	Indeterminate	145	20	36	White	A
Parsa	3.2	117	3	Semi-determinate	148	22	37	Purple	A

a The cultivars are resistant to grain shedding and lodging. M, G, A, L, and K are Mazandaran, Gorgan, Ardabil, Lorestan, and Khuzestan provinces, respectively.

Although the availability of Iranian soybean germplasm is considerable, the existence of these narrow divergent soybean varieties could not respond to high demand for oil and protein content in food ration of humans and livestock in the country. Thus, the industries of oil and livestock/poultry production heavily depend on soybean seed and soybean meal imports. On the other hand, as far as major soybean sales in the domestic market are disrupted or exchange rates for its exports and imports are reduced, soybean seed imports become more and more important. Indeed, until the preconditions for scientific, principled, and optimized soybean cultivation exist, its market shortages must be compensated through soybean imports [7].

In Iran, various factors are involved in the bulk sales of soy as a nutritious grain. Its sale price is one of the key reasons, and its changes are related to fluctuations in its supply and demand in the market. It should be noted that the price of soybean seeds indirectly affects the final price of this agricultural product. According to Table 2, the amount of imported soybean seed and meal was approximately 757,864 (with a value of 561,899,536 $) and 277,094 t (with a value of 161,094,384 $), respectively, in the first four months of 2022. This report has exhibited a reduction of 21.5% of imported soybean seed and 63.7% of imported soybean meal compared to a similar period in 2021 (Table 2).

**Table 2:** Imports of agricultural inputs in the first four months: a comparison with the previous year's similar period.

Row	Product	The first four months of 2022		The first four months of 2021		Percentage of variation	
		Weight (t)	Value ($)	Weight (t)	Value ($)	weight	value
1	Corn	2,330,120	917,012,982	3,145,691	1,040,125,821	−26	−12
2	Maize	274,553	101,039,794	1,492,423	431,726,546	−82	−77
3	Soybean meal	277,094	161,094,384	764,789	411,978,512	−64	−61
4	Soybean seed	757,864	561,899,536	967,778	621,470,815	−22	−10
Total		3,639,631	1,741,046,696	6,370,680	2,505,301,694	−43	−31

The extraction rate of soybean meal in the first four months of 2022 is equal to 660 thousand tons and in the first four months of 2021 is equal to 774 thousand tons.

On the one hand, this reduction rate of import could benefit farmers to encourage them to grow soybeans. On the other hand, in recent years, the guaranteed purchase price for soybean oilseeds per kilogram has been estimated at 330,000 rials, showing that the low price of its sale does not bring economic benefits for farmers compared to higher expenses that are spent on its field preparation, agricultural inputs (fertilizers, pesticides, and herbicides), and other necessary facilities. Therefore, other agronomical crops may overcome soybean cultivation, and soybeans may lose their position in competition with other crops. If the market volume is saturated, it will be in the interest of farmers. This means profitability for those involved in soybean cultivation [8].

Thus, soybean improvement with regard to its yield and production is of great importance in Iran due to its nutritional and economic positions throughout the community.

With respect to importance of this valuable crop for sustainable agriculture and existence of suitable climatic conditions in Iran, this review research was focused on tracking the several challenges and restrictions directly and indirectly involved which leads to lag of this country for production of soybean, following by provision of suitable solutions to improve the contemporary situation of soybeans in the region.

## 2 Constraint to soybean production in Iran

### 2.1 Unbalanced marketing

Soybean trade is considered the most significant obstacle to developing oilseed cultivation in Iran. From a commercial perspective, if the guaranteed purchase price of soybeans is commensurate with production costs and increases in world prices, and payments are made to farmers in a timely manner, similar to wheat, farmers will be encouraged to cultivate soybeans, and its area under cultivation will expand [9]. In northern provinces, where the primary competitor of soybeans is rice, it is believed that soybean prices should be determined in a way that reduces farmers' tendencies to cultivate rice or even reaches equilibrium. Rice cultivation causes several difficulties for ecosystems due to its abundant water needs (rice and soybean water needs are estimated at approximately 800–1200 and 400–550 mm, respectively), leading to adverse environmental and economic effects on the country in the long term [10].

Soybean plays a crucial role in promoting sustainable agriculture in Iran due to its lower water requirements compared to rice [11], which can have adverse environmental and economic effects in the long term.

## 3 Biotic stresses

### 3.1 Common soybean weeds in Iran

Integrated weed management (IWM) is a key method for reducing weed interference in soybean production while maintaining acceptable crop yields and environmental, social, and economic health [12]. To prevent weed damage, knowledge of the critical period of weed competition with soybean is essential for growers to implement effective and timely weed management actions. For instance, young soybean seedlings are not able to compete with many fast-growing tropical weeds [13]. Weeds identification and determination of their density per unit area are basic principles of weed management [14].

In Iran, common weeds in soybean fields usually appear during summer cultivation. Some weeds, such as cockspur grass (*Sorghum halepense*) [15], *Cyndon dactylon* [16], *Setaria viridis* [17], and perennial sow thistle (*Sorghum halepense*) [18], belong to the Graminaceae family.

In addition, some species of nut grass as *Cyperus rotundus* and *C. esculentus* have been reported in some soybean fields [19]. The most important weeds in soybean fields, depending on the region of Iran, are shown in Table 3.

**Table 3:** Major dicotyledonous weeds in soybean fields of Iran.

Weed type	Province				Reported by
	Mazandaran	Ardabil	Khuzestan	Golestan	
Cottonseed (*Abutilon theophrasti*)	×	×	–	×	Bararpour and Abdollahi [20], Salehian and Najafian [21]
Pig weed (*Amaranthus* spp.)	×	×	–	×	Mirshekari et al. [22]
Common cocklebur (*Xanthium strumarium*)	×	×	–	×	Mortezapour et al. [23]
Melon (*Cucumis melo*)	×	–	–	×	Sohrabi et al. [24]
Milkweed (Euphorbia sp.)	×	–	–	×	Ghotbi et al. [25]
Field bindweed (*Convolvulus arvensis*)	×	–	–	–	Sohrabi et al. [24]
Flower-of-an-hour (*Hibiscus trionum*)	–	×	–	–	Zeidali et al. [26]
*Physalis divaricate*	–	–	×	–	Alam et al. [27]
Bishop's flower (*Ammi majus*)	–	–	×	–	Khan et al. [28]
Sea beet (Beta maritime)	–	–	×	–	Masoumi et al. [29]
*Ipomoea* sp.	–	–	–	×	Siahmarguee et al. [30]

The methods of controlling weeds vary based on the area under soybean cultivation and the type of weeds present. However, suggestive instructions are available for farmers to control weeds and achieve proper performance. Rapid soybean growth is one approach to controlling weeds if the plants are regularly green. Tillage cultivation is another common method to not only control weeds but also break up tubers and improve soil aeration. Higher plant density and narrower cultivation rows can also allow soybean plants to compete better with weeds. Herbicides, as an effective and immediate technique, are another weed control method that can be applied pre- and post-emergence [30]. Table 4 indicates the list of herbicides used in soybean fields based on their type and applicable time of usage. Environmentally-friendly and natural methods for managing soybean weeds in Iran include crop rotation, cover cropping, manual weeding, and mulching [31]. These approaches can effectively reduce weed pressure while supporting soil health and minimizing negative environmental impacts. Intercropping with compatible plant species can also enhance biodiversity and suppress weed growth in soybean fields [32].

**Table 4:** Common herbicide used in soybean fields of Iran.

Public name	Business name	L/ha	Method of usage	Type of weed
Bentazon	Basagran	2	4-6 leaves of weed	Broad leaf
Clethodim	Select super	1	4-6 leaves of weed	Narrow leaf
Haloxyfop - R Methyl Ester	Gallant super	0.75–1	4-6 leaves of weed	Narrow leaf
Quizalofop-*p*-tefuryl	Pantera	1–1.5	4-6 leaves of weed	Narrow leaf
Trifluralin	Treflan	2–2.5	Pre-planting (mixture with soil)	Broad/narrow leaf
Ethal floralin	Sonalan	3–3.5	Pre-planting (mixture with soil)	Broad/narrow leaf
Di-nitramin	Cobex	3	Pre-planting (mixture with soil)	Broad/narrow leaf
Metribuzin	Sencor	600 gr	After soybean cultivation-before soybean and weed emergence	Broad/narrow leaf
Imazethapyr	Pursuit	0.75–1	After soybean cultivation-before soybean and weed emergence	Broad/narrow leaf
Fomesafen	Reflex	1–1.5	4-6 leaves of weed	Broad leaf

### 3.2 Frequent soybean diseases in Iran

Soybean is attacked by approximately 100 pathogens worldwide, of which 40% are economically significant, causing a 10–30% reduction in plant yields [33,34]. The severity of infection and disease damage depends largely on environmental conditions, such as temperature, humidity, soil type, weeds, disease-carrying insects, and lack of attention to crop rotation and planting of disease-sensitive cultivars [35]. Charcoal rot and root and shoot rot diseases, caused by *Macrophomina phaseolina* and *Phytophthora sojae*, respectively, are the most significant among the various fungal pathogenic agents affecting soybean [36]. Charcoal rot in soybean was first reported in 1986 in Mazandaran and Khuzestan provinces, with infection rates increasing by 25–95% in subsequent years [37]. Symptoms of the disease include poor seed germination, conversion of green color of root vascular tissue to brown, darkening of the collar, plant paleness, premature aging, and wilted plants [38]. The agent of this disease is related to seed and soil, and its survival in soil and plant residues is similar to that of sclerotia. Factors contributing to the occurrence of this disease include heat and drought stress, cultivar sensitivity, disease inoculum, cultivation date, and row distance [39].

Strategies to control this disease include the use of healthy seeds, prevention of water stress, intercropping, biological control, and tolerant varieties. Tolerant varieties such as Sari, Nekador, Katol, and Caspian have been introduced for the Mazandaran province due to their high seed yield [40,41].

Another common soybean disease is root and shoot rot, which was first reported in Lorestan province in 1998, causing seed rot and plantlet death [42,43]. Symptoms of this disease include rotting of secondary roots, soft and bruised shoot infections, yellowish leaves, and plant death. To manage this disease, appropriate drainage, proper intercropping, use of resistant cultivars, biological control, and use of fungicides are recommended [44]. Currently, the early-maturing cultivar Kosar has been recognized as resistant to *Phytophthora* in Iran [45].

Sustainable and organic methods for controlling soybean diseases include the use of disease-resistant cultivars, crop rotation, intercropping, and biological control agents. Proper soil management practices, such as maintaining appropriate drainage and avoiding water stress, can also help prevent the spread of diseases. In addition, regular field monitoring and early detection of diseases can aid in timely treatment with organic fungicides or other natural treatments. These approaches promote a healthy and resilient agroecosystem while minimizing environmental impacts [46].

### 3.3 Customary soybean pests in Iran

Pests can cause a reduction in leaf area, number of pods, and number of seeds in soybean plants, leading to a decrease in seed yield and quality [47]. Insects, worms, larvae, and mites are the most important pests that affect soybeans. To accurately diagnose and identify pest damage, it is important to have knowledge of the entire biological cycle of each pest and its associated damage [48].

The main pests of soybean fields have known such as *Spodoptera exigua* (*Caraderina*), *Tetranychus urticae*, *Bemisia tabaci*, *Agrotis* spp. and *Helicoverpa armigera* which could reduce the plant production by 70–80% [49]. Two-spotted spider mite (TSSM; *Tetranychus urticae* Koch), is one of the most important pests which its population has increased enormously in the different soybean-producing areas of Iran and worldwide [50]. Also, it was reported the optimal temperature for mated females laid eggs of *T. kanzawai* Kishida (*Tetranychidae*) was 25 °C (237.96 eggs), however, by increasing the temperature by 37.5 °C, it was reduced by 30.54 eggs [51].

A previous study reported that, among 14 soybean genotypes, the Dpx and Sahar genotypes were the most resistant to the Two-Spotted Spider Mite (TSSM) based on the pest's life cycle and fecundity [48]. In addition, it was found that some soybean varieties, such as M9, Williams, Clark, L17, M7, M4, and Zane, were suitable host plants for reproduction of *Helicoverpa armigera*, while others, including DPX, Clark, 356, M4, M7, M9, Gorgan3, Sahar, and Williams, were less suitable [52]. In another study, the digestive proteolytic and amylolytic activities of *H. armigera* were evaluated in response to feeding on different soybean cultivars, including 356, M4, M7, M9, Clark, Sahar, JK, BP, Williams, L17, Zane, Gorgan3, and DPX. The authors reported that L17 and Sahar cultivars were partially resistant to this pest, possibly due to the presence of secondary chemicals or proteinaceous protease inhibitors [53].

The Beet Armyworm (*Spodoptera exigua Hübner*) is another serious pest that is found in soybean fields in Iran [54]. In this case, it was reported that nine soybean cultivars based on the biological parameters of *S. exigua*, were classified in two main groups. The authors showed that the first group was consisted of susceptible cultivars i.e. 033, Zan, M4, TMS, and M11, whereas the resistant cultivars i.e. 032, M7, M9 and Hill were in the second group [54].

Furthermore, another study has shown that the whitefly (*Bemisia tabaci*; Hemiptera: Aleyrodidae) can cause economic damage to several crops, including soybean, by extracting large amounts of sap from the phloem and negatively affecting plant growth and development. In addition, the sweetpotato whitefly, *B. tabaci*, is currently a devastating pest in tropical and subtropical countries due to its role in transmitting various plant viruses [55].

To manage and control pests in soybean fields, non-chemical methods such as winter water freezing, fall plowing, and crop rotation could be effective and beneficial. However, if chemical control is necessary, it should be used as a last resort due to its potential side effects [56]. In Iran, based on the common pests found in soybean fields, plant protection researchers recommend the use of chemical treatments as shown in Table 5.

**Table 5:** The common pest in soybean fields of Iran and pesticides usage.

Pest name	Recommended pesticide	Usage per ha
*Spodoptera exigua*	Avant (Indoxacarb)	250 ml
	Larvinr (Thiodicarb)	1 kg
	Matrin	500 ml
(Egyption cotton leaf worm) *Spodoptera littoralis*	Avant (Indoxacarb)	250 ml
	Atabron (chlofloroazoron)	1000 ml
	Matrin	500 ml
*Helicoverpa armigera* Hubner	Avant (Indoxacarb)	250 ml
	Atabron (chlofloroazoron)	1000 ml
	Larvinr (Thiodicarb)	1 kg
*Tetranychus urticae* Koch	Neuron	1000 ml
Thrips	Dimethoate	1000 ml

Sustainable agriculture methods for controlling soybean pests include the use of biological control agents, such as predators and parasitoids, as well as the promotion of beneficial insects through habitat management practices. In addition, the use of resistant soybean cultivars, crop rotation, and intercropping can help reduce pest populations and prevent outbreaks. Proper soil management practices, such as maintaining optimal fertility and moisture levels, can also promote plant health and resilience to pest attacks [57]. These approaches minimize the use of synthetic pesticides and promote a healthy agroecosystem.

### 3.4 Pod abnormality in soybean fields of Iran

In recent years, a new disorder in soybean, called "pod abnormality" or "pod dissertation syndrome," has been reported in the north of Iran, causing reductions in soybean yield [58]. This disorder was first reported in Golestan province in 1985 and is observed with varying intensity every year, making it one of the most significant problems of soybean in the province. In 2011, the incidence of pod abnormality was reported on a large scale, affecting 18% of the total soybean area under cultivation in Golestan province. Four years later, in 2015, approximately 39% of the total soybean cultivation area in Golestan province was infected to various degrees [59]. In addition, similar symptoms of this phenomenon have appeared in neighboring provinces, such as Mazandaran [60].

**Fig. 3:**
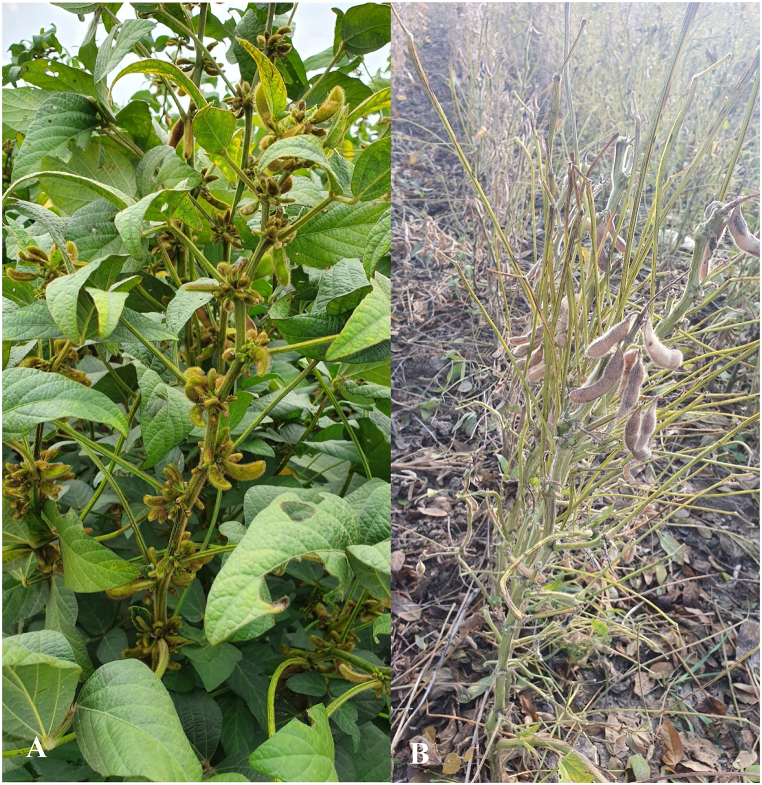
Symptoms of soybean pod and green stem disorder in North of Iran. a) The reproductive stage of R4 (Full pod formation) and R5 (The beginning of seed fill), b) The reproductive stage of R8 (Full maturity).

The symptoms of this disorder can be observed on a large scale or in some spots of soybean fields. They appear at the beginning of the flowering stage, including the loss of blooms, which results in the loss of capsules in the whole or some areas of the plant, the appearance of sickle-shaped capsules, shiny leaves with dark green color, and a forage state of the plant (Fig. 3a and b). In addition, soybean plants continue their vegetative growth more than usual, but flowers dry out without conversion to capsules [61]. In some cases, a similar syndrome called “green stem” (GSD) occurs, resulting in mature pods and seeds hanging from green stems, which can make harvesting difficult. The causes of these disorders are not clearly known but could be attributed to environmental stress, high summer temperatures, herbicide drift, severe drought, genetic mutation, or an imbalance in the carbon source-sink ratio [62].

Recent studies have suggested that phytoplasmic agents could be a cause of this phenomenon. The responsible phytoplasma belongs to the group of 16SrVI as *Candidatus phytoplasma* trifolii which has different hosts in Iran such as alfalfa [63], willow [64], *Calendula officinalis* [65], cucumber [66], date palm [60], peach [67], and Catharanthus [68]. It was reported that the isolate of soybean pod abnormality agent had the most similarity with disease agents of yellowish willow, big bud of tomato, and potato proliferation.

Molecular analysis reports indicate that bugs infected with *Phytoplasma* were collected from soybean fields in Iran. Willow trees and *Phragmites australis* with stripped-yellow symptoms have been identified as winter shelters and bug transporters of this disease. In a previous study, *Creontiades pallidus*, a bug, was indicated to have the capability of transferring disease agents from infected hosts surrounding soybean fields to the main soybean farm. In addition to specific symptoms of virus diseases caused by tobacco and tomato ring spot virus, Arabis mosaic virus and tobacco strip virus may also result in a lack of capsulation in soybean [69]. Another study showed that the *Hemiptera Creontiades pallidus*, known as the cotton shredder bug, had the capability to transmit the *'Candidatus Phytoplasma trifolii*' strain to healthy soybean plants under insect-proof conditions using ELSA and nested PCR amplification for virus and phytoplasmic detection, respectively [70].

Numerous researchers have aimed to develop more effective strategies to manage this disease in soybean field conditions. Recent research has shown that the use of insecticides such as dimethoate and acetamiprid can effectively control the bug population when accompanied by micro-fertilizers (such as boron and zinc) to stimulate flowering and pod production. Another method for controlling *Phragmites australis* surrounding soybean farms is to avoid planting willow trees in neighboring areas. Although the transmission of the disease agent by soybean seed is less than 2%, separating clear seeds from diseased ones is an effective approach and highly recommended.

## 4 Abiotic stresses

### 4.1 Drought stress

Drought stress is a critical factor that limits soybean yield and crop production by up to 40 percent [71,72]. The main causes of drought are low humidity in the atmosphere, high temperature, and water deficiency [73]. This stress inhibits cell division and growth processes [74], resulting in the closure of stomata and reduction of CO2 flow into mesophilic cells [75], as well as inhibition of ribulose 1 and 5 bisphosphate regeneration, protein and Rubisco enzyme activity, optical phosphorylation, and ATP synthesis. Ultimately, drought stress decreases the yield and quality of plants [[76], [77], [78]].

Dehydration also reduces plant access to nitrogen, nitrogen uptake, and the activity of nitrogen-fixing enzymes, mainly nitrate reductase and glutamine synthetase [79]. Because plant photosynthetic capacity is related to leaf nitrogen, limitations in photosynthesis and growth due to environmental stresses such as drought are probably the result of changes in nitrogen levels and plant accessibility to this element [80]. The effects of drought stress on plant physiological processes have been extensively studied, including the effect on growth [77], gene expression [81], signal pathways [82], photosynthesis [83], and mitochondrial respiration [84].

Iran is considered one of the dry and semi-arid countries worldwide. In recent years, drought stress and rainfall decrease have caused severe losses in the country's agricultural sector, even in the northern part of Iran with high yearly rainfall. Over half of the people in Golestan and Mazandaran provinces rely on agriculture, which has been affected by the slump in the agriculture sector. Therefore, the cultivation of crops with low water needs is exceptionally necessary to conserve water sources [85].

Soybean is one of the strategic crops, specifically in these provinces, due to its high compatibility with weather conditions, less need for water than rice (as the dominant cultivated crop), reinforcement of agronomical soil, and reduction of soil erosion, as well as stabilizing ecosystems. Indeed, soybean could be an appropriate replacement for rice (being a main competitor), emphasizing farmers’ interest in cultivating this valuable crop. However, soybean yield reduction has occurred in several consecutive years, which is attributed to heat and drought [86]. For example, Guimaraes-Dias et al. [87] reported a remarkable decrease in soybean production, estimating less than 69% of its actual capability. Moreover, long-term drought during the summer season led to a reduction in soybean seed yield in Brazil, the second-biggest soybean producer worldwide.

In order to cope with drought stress, plants show a variety of morphological, physiological, biochemical, and molecular responses at different levels [88,89]. Responses to drought stress at the cellular level include membrane system configurations, changes in cell wall architecture, changes in cell cycle and cell division, and production and accumulation of compatible metabolites (such as proline, raffinose, and glycine betaine). Under stress conditions, redox metabolism of cells is activated to remove excess amounts of reactive oxygen species (ROS) and re-establish cellular redox equilibrium [90]. At the molecular level, the expression of some genes, including genes involved in protection and direct response to stress, such as osmo-protectants, enzymes involved in detoxification, transporters, and regulatory proteins containing transcription factors, protein kinases, and phosphatases, are induced and stimulated [91]. Identification of these adaptation pathways under drought stress conditions in plant species, especially susceptible and tolerant cultivars, is tremendously important because they can be used as markers in plant breeding programs to expand drought-resistant plant cultivars.

In recent years, the effects of drought on different mechanisms of soybean have been extensively reported [92]. It is indispensable demand for identification of the mechanisms associated with drought tolerance to select resistant and adapted cultivars to increase soybean yield. Maleki et al. [93] indicated that drought stress caused the lowest seed yield during the stages of seed filling (2682 kg.ha^−1^) and flowering stage (2918 kg.ha^−1^), followed by grain filling stage as the most sensitive reproductive stage in soybean under drought stress. Navabpour et al. [94] evaluated the effects of drought stress on important agronomic traits, protein and oil content of soybean, reporting that PE10 and DS2 genotypes had the highest grain yield and the variety of Sari had the lowest grain yield under drought stress condition. In another study, Kargar et al. [95] reported that the varieties of Clark, LD9, and Elgine had the highest grain yield under drought stress condition. Additionally, the Nekador soybean cultivar was reported to be drought-tolerant, with other appropriate agronomic traits such as its proper height, resistance to lodging and shattering, as well as high yield [41].

Besides the aforementioned approaches, conservative or no-tillage cultivation could be an effective tool to increase soybean yield under drought stress. Numerous studies have been carried out on the effect of tillage strategies on soybean yield [96,97]. It has been shown that tillage is not a significant management factor to increase soybean yield, as other factors, including planting date, row spacing, and cultivar selection, are of considerable importance. The key issues of whether tillage is important are to have a good understanding of field conditions in terms of drainage and soil-borne pathogens [98]. Understanding these conditions is essential for determining the need for tillage in cropping systems. In one study, it was indicated that even with improved plant genetics, there has been basically no consistent change in response to tillage than no-tillage systems, which lead to an increase in seed yield [99]. It was reported that current planter's machines and herbicides now allow us to plant and control weeds without tillage practices, and variety selective-fungicide seed treatment can further enhance the profitability of no-tillage systems [100].

Iran is facing the devastating impacts of climate change, including drought and water scarcity, which have severely affected its agricultural sector [101]. With over half of the population in Golestan and Mazandaran provinces relying on agriculture, the need for sustainable agriculture practices and conservation of water resources is crucial. Soybean, a strategic crop in these provinces, has been identified as a potential solution due to its low water requirements and ability to reinforce agronomical soil and reduce soil erosion. Cultivating soybean instead of water-intensive crops like rice can conserve water resources and build a more sustainable agricultural system in Iran.

### 4.2 Salt stress

Around 50% of the world's arable land is affected by salinity [102]. After drought, salinity is one of the most significant and pervasive environmental stresses worldwide [103]. Due to Iran's location in arid and semi-arid climate zones, nearly 50% of the cultivated land is affected by varying degrees of salinity and alkalinity [104]. In these regions, salinity is a major obstacle to the production of agronomic and horticultural crops [105]. Salinity is a comprehensive issue in Iran that limits sustainable agricultural production. Various parts of the country's dry and semi-arid regions, particularly the central plateau, southern coastal plains, and Khuzestan plains, are affected by different levels of salinity [106].

Plants are generally classified as either halophytes (salt-tolerant) or non-halophytes (salt-sensitive) based on their growth response to salt concentrations. Halophytes are the main plants found in saline areas, while non-halophytes are unable to tolerate salinity [107]. The growth response of plants to salt stress is complex and highly dependent on various factors, including stress intensity, cultivar type, plant species, plant development stage, and stress duration [108]. Salinity-tolerant plants must possess the ability to adjust osmotic pressure, which occurs approximately once a day. Plants can often adjust to reduced water potential without losing water, except when salt levels are high [109].

Under salt stress conditions, plant growth is reduced due to a decrease in water potential in the root growth environment or the specific effects of ions on metabolic processes. The most apparent effect of reduced plant growth under salt stress is a decrease in leaf area [110]. Even if the rate of photosynthesis per unit area of leaf remains constant, the rate of growth of the whole plant will be reduced due to a decrease in photosynthesis [111]. Furthermore, sodium chloride salinity affects the transport of water and ions in plants, which can result in changes in the ionic balance and nutrient status of the plant. Salinity affects both vegetative and reproductive growth, which can lead to a decrease in dry weight and plant yield [112].

Since solving and overcoming the problem of soil salinity requires a long-term and time-consuming effort, it is essential to focus on soil amendments and correction of plant physiological processes to improve their performance under salt stress conditions [113]. One way to combat this issue is to improve soil drainage and water control [114]. Although these methods can reduce the amount and extent of salt levels in saline soils, the introduction of new strategies is crucial due to high engineering costs and management requirements [115]. Another strategy is to identify crops that are tolerant to salt.

Among the various crops tested, legumes are generally found to be more sensitive to salinity [116]. Soybean is considered a moderately salt-tolerant crop plant, with a threshold of 5 dS/m. However, salt-sensitive soybean cultivars were severely affected under soil salinity levels of 8 dS/m, resulting in no seed production [117]. The reason for the reduction in seed yield could be attributed to reduced germination, low seedling emergence, poor plant growth and development [118,119], as well as negative effects on seed protein, oil, and carbohydrate content [120].

Numerous studies have investigated the effects of salt stress on various characteristics of soybean. There is considerable interest in finding eco-friendly and cost-effective supplements to improve plant growth and productivity, as well as to minimize the detrimental impacts of physicochemical stresses on plant growth, development, and metabolism. For example, the use of chitosan to alleviate the harmful effects of salinity on soybean seed germination was investigated, and the results suggested that a 0.25% w/v chitosan treatment is ideal [121]. Furthermore, the simultaneous application of selenium and salicylic acid may be a promising approach to alleviate the toxicity associated with salinity [122]. Seed invigoration with Trichoderma harzianum was also found to mitigate the adverse impacts of salinity on soybean seedlings [123]. Kamrava et al. [124] reported that the Hill CE variety is the most tolerant, while Ford and 032-240-D are sensitive varieties.

Iran is at risk of salt stress due to its geographical location. It has been estimated that approximately 27 million hectares of Iran's land (around 17%) are exposed to salt stress [125]. Therefore, it is essential to establish a compatible agricultural system that can withstand salt stress. This requires effective salt stress management, including various approaches such as soil drainage, leaching methods, and the implementation of breeding programs with the objective of creating salt-tolerant lines/cultivars.

Climate change and global warming are affecting crop production and food security in Iran. Sustainable agriculture practices such as conservation agriculture, the use of climate-smart crops like soybean, and precision agriculture techniques can mitigate the impacts of climate change on agriculture. Soybean, a moderately salt-tolerant crop, can be grown with eco-friendly and cost-effective supplements such as chitosan, selenium, and salicylic acid under salt stress conditions. The cultivation of soybean and the implementation of effective salt stress management strategies such as soil amendments, leaching methods, and breeding programs to create salt-tolerant soybean cultivars can be a sustainable solution to the issue of salinity in Iran. These practices can contribute to food security, environmental sustainability, and the livelihoods of farmers and rural communities.

### 4.3 Considered as secondary crops

Soybean occupies a significant place in the agriculture industry due to its production of oil and forage supply, and reducing imports. Therefore, expanding soybean cultivation areas should be prioritized among other oil crops [126]. To overcome the challenges faced by soybean cultivation, various aspects such as expanding its production area, reducing input costs, increasing purchase price, and using optimal soybean cultivars can be considered [127].

However, in the northern part of Iran, rice is the main competitor of soybean, and it is cultivated twice a year. Farmers prefer to plant rice, especially if there is enough water for agriculture, due to its higher sale price, better market, and profitability. Furthermore, in rain-fed conditions, farmers tend to cultivate cereal crops because of their higher stability in production and lower losses in storage compared to soybean [128]. Additionally, soybean yields are not assured, and it is more sensitive to diseases, pests, and weather conditions than cereals. As a result, it is considered a second crop and fails to compete with other crops [129].

Despite these challenges, soybean has several advantages that make it a viable main crop. It has high nutritional value for both humans and livestock, enhances soil maintenance and prevents erosion, has a short growth period, does not interfere with planting conventional autumn crops in the region, and has economic benefits that create job opportunities [130]. Overall, expanding soybean cultivation can lead to economic benefits and job creation, as well as improving soil health and providing a valuable source of nutrition. However, to overcome the current challenges, it is necessary to develop optimal soybean cultivars and to implement effective management strategies to increase soybean yields and profitability.

### 4.4 Limited agricultural equipment

Agricultural mechanization is an essential component of agricultural development, playing a critical role in responding to the diverse needs of soybean producers and consumers [131]. Due to limited arable land in Iran, increasing soybean yield per hectare is crucial for enhancing agricultural productivity [132]. Proper crop residue management is an essential principle of agricultural production systems, as it has positive effects on plant performance, energy consumption, and soil and environmental conservation [133].

Various factors, including climatic conditions, cultivation date, plant population, and agronomic management practices, affect soybean yield [134]. Studies on different plowing techniques after maize cultivation have demonstrated no significant difference in soybean yield [135]. However, soybean seed yield was higher under normal pillow conditions than conservative tillage methods, and the yield varied based on the type of plant cultivated before soybean [136]. Additionally, conventional tillage with complete previous crop residue resulted in the highest soybean yield [137].

In Iran, soybean yield is estimated to be 1.5–2.5 t/ha under rain-fed/irrigation conditions, respectively. However, the actual yield of introduced soybean varieties is lower than the estimated yield due to the lack of proper mechanization practices. The challenges facing soybean production in Iran include a lack of machinery and agricultural implements, inadequate distribution of agricultural machinery in different regions, outdated tools and machines, time gaps between demand and supply of machinery through the banking system, insufficient technical knowledge among researchers and farmers on using machines and agricultural implements, and weak support services for repairing and maintaining machines [138]. Furthermore, the lack of necessary machinery and tools such as trailers, seed drills, planters, sprayers, and narrow-wheel tractors for mechanized operations, as well as the lack of heads for soybean harvesting, increase the difficulty of soybean cultivation and its costs, leading to a decline in the desire of farmers to grow this crop [139].

Therefore, timely and appropriate use of vehicles for soybean cultivation, including conservative tillage/no-tillage systems, and harvesting could help farmers achieve higher yields and improve soil conditions, prevent soil erosion, conserve soybean-specific bacterial activity, and maintain soil humidity.

Therefore, proper agricultural mechanization practices are essential for enhancing soybean productivity and overcoming the challenges faced by soybean cultivation in Iran.

### 4.5 Soybean genetic diversity

It is crucial to determine the genetic diversity and their associations among breeding materials for crop improvement strategies [140]. To screen out the desired genetic materials, a deep view on a prerequisite of characterization and evaluation of germplasm is indispensable for genetic boost programs. Preparation of germplasm collections depend on availability of several numbers of accessions with genetic materials for the concerned traits [141,142]. Since diversity is the most vital factor for surviving organisms such as plants against changing environment, biodiversity in agriculture has an influential effect on nutritional security of next generation.

Soybean like numerous significant crops is an autogamous species with low percentage of out crossing. Attenuation of genetic diversity has been due to 1) inbreeding resulting from harmful mutations which cause remove the non-harmful alleles at linked loci, and 2) evolutionary events such as domestication, founding events, and selection which effect on the level of sequence variation within a crop [[143], [144], [145]].

The narrow genetic base of soybean could be a major limitation in its breeding schemes because of lack of genetic variability, cultivar susceptibility to pathogens and herbivores, and reaching of yield plateaus [146,147]. Therefore, utilization of soybean germplasm as a natural source and make use of its potential in breeding strategies (as non-hybridization and hybridization) can be effective tool to broaden the current soybean genetic base [148].

In Iran, the old soybean varieties were introduced from foreign countries such as Wiliams, Hill, Zane, Clark and so on, since cultivar introduction is one of the best and quickest approach of breeding program in automatous plants like soybean [149]. By evaluation of compatibility of soybean imported cultivars/genotypes in consideration of their yield and other important traits, there is possibility to cultivate them in different areas. In addition, the recent cultivars of Sahar and Katol which are planted in North of Iran and Khozestan have known as imported cultivars [150].

Although, these imported cultivars cultivate as a compatible agronomical genotype, they will be used as parents for hybridization breeding programs. Numerous studies have been performed according to evaluate the soybean genotypes/cultivars compatibility. For example, the 364 soybean genotypes were evaluated based on morphological traits showing the high phenotypic variations of some traits such as number of capsules in branches, number of branches and null nodules at early maturation [151]. Moreover, the morphological and phonological diversity of 124 soybean imported genotypes were investigated using PCA analysis [152]. Based on their results 32% of variation among the studied genotypes was related to the first principal component, which was correlated by the phonological traits i.e. days to flowering, podding, seed filling and ripening.

Besides of the necessity of soybean imported cultivars assessment based on different agronomical traits, their investigation on the basis of their resistance to pest and diseases are of great importance [153]. Among all soybean diseases, the root and shoot *Phythphthora* rot is one the most common and dangerous soybean diseases around the world even in Iran which cause about 100 percent reduction of soybean performance specially in Lorestan, Golestan and Mazandaran provinces [154]. In this case, Majidian et al. [155] screened and evaluated some imported cultivars and pure lines of soybean for *Phytophthora* rot resistance and agronomical and morphological traits. The authors reported that some of the cultivars i.e. Winchester, Beeson 80, Amcor 89, Beeson, L75-3735, L77-1794, Oak land were resistant to the disease and had a high plant seed yield. Also, their results showed that a high level of variation among the studied cultivars and pure lines based on the measured characters.

In order to expand genetic diversity in soybean and other autogamous species, breeding after hybridization will create genetic diversity specially when there is no/narrow genetic variability in the population [156]. Since, soybean is a typical self-pollinated crop and its natural outcrossing rate is low (0.03–6.32%), the aforementioned method following by pedigree, bulk, single seed descend, back cross, double haploid production will improve its diversity with emphasis on breeding based on low heritability traits/multiple genes traits [157].

In Iran, the current breeding programs of soybean have focused on increasing soybean seed, oil and protein yield as well as creation of tolerance/resistance to various abiotic and biotic stresses. The uses of divergent soybean cultivars/genotypes which cover the vast specific agronomical properties have recommended making achievement in soybean breeding programs. In general, knowledge of genetic variation within soybean germplasm collections is essential to understand the evolutionary relations among accessions and tracing the genes of interest. In this case, molecular markers have key role to identify genetic divergent among soybean accessions [158]. Numerous studies have been performed to evaluate soybean genetic diversity by different molecular markers such as RAPD and ISJ [159], RAPD [160], and DAF [161].

Given the narrow genetic sources of soybean in Iran, investigating soybean genetic diversity and recognizing genotypes with high yield potential and tolerance to environmental stresses is necessary to make progress in further breeding programs. Utilizing appropriate approaches on the basis of morphological, molecular, and biochemical processes, either in isolation or in combination, is of great significance in the field of soybean breeding projects. By utilizing genetic diversity in soybean breeding programs, it is possible to develop new varieties that are adapted to local growing conditions and that can contribute to sustainable agriculture in Iran. Additionally, conservation of soybean genetic resources can ensure the availability of diverse genetic materials for future breeding programs and research endeavors.

Genetic diversity in soybean is crucial for sustainable agriculture in Iran and globally [162]. It provides resilience against biotic and abiotic stresses, adaptation to changing environmental conditions, and a wide range of traits necessary for crop improvement. Maintaining genetic diversity is important for reducing reliance on chemical inputs, improving resource use efficiency, and enhancing agro-ecosystem resilience. The narrow genetic base of soybean in Iran highlights the need to utilize genetic diversity in breeding programs to develop locally-adapted varieties. Additionally, conservation of genetic resources can ensure availability for future breeding and research efforts.

### 4.6 Energy consumption and efficiency in soybean production

Energy consumption and energy productivity are important factors to consider in soybean production in Iran. Efficient energy use ensures optimal resource allocation and reduces dependence on fossil fuels. Improving energy productivity enhances agricultural sustainability and reduces environmental impacts. By optimizing energy inputs, such as electricity, fertilizers, and fuel, farmers can achieve higher yields, enhance food security, and contribute to a more sustainable and resilient soybean production system in the country.

Several studies conducted in Iran have examined these aspects and provided valuable insights. Abbas and Majid [163] emphasized the need for domestic soybean production in Iran to achieve food security and reduce reliance on imports. Their study highlighted the importance of improving energy consumption efficiency, increasing the use of renewable energy, and implementing sustainable practices such as early sowing and proper rotation. Alimagham et al. [164] analyzed energy flow, greenhouse gas emissions, and efficiency in soybean production in Gorgan, Iran. They found that conventional scenarios had higher energy efficiency and lower greenhouse gas emissions compared to mechanized scenarios. Enhancing energy efficiency, particularly in mechanized scenarios, was identified as a potential means to improve environmental friendliness in soybean production. Mousavi-Avval et al. [165] identified electrical energy as a significant contributor to energy consumption and suggested potential energy savings. The study highlighted the importance of energy optimization in soybean production. Ramedani et al. [166] revealed diesel fuel, chemical fertilizers, and irrigation water as the main energy inputs. They also conducted sensitivity analysis, which showed the positive impact of seed energy on yield. Mousavi Avval et al. [167] identified irrigation, tillage, and harvesting as significant energy-consuming operations and suggested improving energy efficiency and adopting sustainable practices to enhance the environmental friendliness of oilseed production. Rajaeifar et al. [168] focused specifically on biodiesel production from soybean in Golestan province. Their analysis of the life-cycle process highlighted favorable net energy gain and low greenhouse gas emissions. The study underscored the potential of soybean-based biodiesel for enhancing energy security, while cautioning the need to consider food production competition.

The literature review emphasized the importance of energy consumption, energy efficiency, and sustainable practices in soybean production in Iran. They provide valuable insights into the optimization of energy inputs and their impact on yield, as well as the potential for enhancing environmental friendliness in soybean production.

### 4.7 Oil-seeds mafia

The Oil-seeds mafia in Iran is one of the main factors preventing the development of oil production and self-sufficiency in this field. This mafia consists of individuals and groups who benefit from the import of oilseeds, and by controlling prices within the country and creating sanctions and obstacles, they weaken domestic production and illegally profit from oil-seeds imports. This mafia is supported by some officials and individuals in powerful government positions as well as in the private sector, and they strengthen themselves through corruption and illegal activities. They create problems for domestic production through sanctions and economic pressure, increase oil imports to maintain their interests, and control prices within the country, making it difficult for domestic production to compete with the price of imported oil. To prevent the influence of the oil mafia and promote self-sufficiency in oil production in Iran, the following solutions can be implemented:

1. Implementation of guaranteed purchase law for agricultural products: This law can incentivize producers and ensure that the minimum price they receive for their products is fair. This measure can strengthen domestic production and reduce dependence on imports.
2. Reduction of customs tariffs: The Ministry of Agricultural Jihad can encourage domestic production and facilitate competition with imports by reducing customs tariffs on oilseed imports.
3. Strengthening government support: The government can support producers by creating specific policies and programs for the development of oil production and creating a favorable environment for the growth of the oil extraction industry.
4. Technical advancements and training: By providing training on up-to-date techniques and improving technical aspects of oil production and optimizing production processes, producers can enhance the quality and efficiency of their production and improve competitiveness.
5. Promoting research and development: The government can encourage research and development in oil-seeds production and related technologies by providing facilities and financial support. This action can lead to process improvements and cost reductions.

These solutions can significantly improve the oil-seeds, especially the development of soybean production in Iran, and enable self-sufficiency in oil-seeds production. However, for the success of these solutions, it is necessary for the government and other relevant institutions to take decisive and effective actions in decision-making and implementation in this field and prioritize the fight against the mafia.

### 4.8 Advantages of soybean farming in sustainable agriculture in Iran

Soybean is an important source of animal feed in Iran, yet its cultivation is not widespread in the country, posing a significant challenge to sustainable livestock production. The import of soybeans from other countries further exacerbates the issue, making the sustainability of animal husbandry highly vulnerable. To promote sustainable livestock production in Iran, it is essential to increase the cultivation of soybean in the country, which could lead to the flourishing of animal husbandry and an increase in sustainability in Iran's agricultural sector.

Soybean cultivation has the potential to provide numerous benefits for sustainable livestock production in Iran. Soybean is a high protein crop with a wide range of nutritional benefits that can improve animal health and productivity [169]. Soybean meal, a byproduct of soybean processing, can be used as a replacement for costly imported feed, reducing production costs and increasing profitability for farmers [170].

Due to climate change, extreme weather events such as droughts have become more frequent and intense in many parts of the world [171]. Iran is no exception, the country has faced severe droughts and water shortages in recent decades [172]. Prolonged droughts have led to increased soil salinity which poses a grave threat to agricultural production and food security [173]. Therefore, alternative cropping systems and drought-tolerant crops are urgently needed to ensure sustainable agriculture under such harsh conditions.

Soybean is a promising crop that can thrive under drought and soil salinity [174]. The plant is considered a less water-intensive crop compared to rice as a major crop in Iran. Therefore, replacing rice with soybean can conserve water resources and improve resilience to drought. In addition, soybean has a deep root system that can extract water from deeper soil layers, enabling it to tolerate drought better than shallow-rooted crops [175].

Furthermore, the deep roots of soybean can help improve soil structure, decrease soil compaction, and reduce soil erosion [176]. This is particularly important for saline soils as it can improve drainage and leach sodium salts below the root zone.

Soybean plays an important role in sustainable agriculture in saline soils due to its ability to fix atmospheric nitrogen [177]. As a leguminous crop, soybean forms a symbiotic relationship with nitrogen-fixing bacteria known as rhizobia. The rhizobia live in nodules on the roots of the soybean plant, where they convert atmospheric nitrogen into a form that can be used by the plant. This helps to provide a source of nitrogen for the soybean crop, enhancing its productivity and sustainability in saline soils.

In addition to fixing nitrogen, soybean cultivation can also help to improve soil structure and reduce soil salinity. Soybean residues left in the soil after harvest can provide a source of carbon and nitrogen for soil microorganisms, including nitrogen-fixing bacteria, which can enhance soil fertility and reduce the need for synthetic fertilizers. Furthermore, the use of cover crops in soybean fields can help to increase the diversity of microbial communities in the soil, which can further enhance soil fertility and sustainability [178].

The use of soybean in rotation with other crops can also help to enhance the abundance and diversity of nitrogen-fixing bacteria in the soil [179]. This can help to promote sustainable agriculture in saline soils by reducing the need for synthetic fertilizers and enhancing soil fertility. By promoting the use of soybean in sustainable agricultural practices, it is possible to enhance crop productivity, reduce the environmental impact of agriculture, and promote long-term sustainability.

Soybean cultivation can provide several environmental benefits, including reducing greenhouse gas emissions, improving water quality, and promoting biodiversity. One way in which soybean cultivation can reduce greenhouse gas emissions is through its ability to fix atmospheric nitrogen. Synthetic nitrogen fertilizers are a significant contributor to greenhouse gas emissions, as their production and use require high amounts of energy and release nitrous oxide, a potent greenhouse gas, into the atmosphere [180]. By fixing nitrogen in the soil through the symbiotic relationship with rhizobia, soybean cultivation can reduce the need for synthetic nitrogen fertilizers, thereby reducing greenhouse gas emissions.

The plant cultivation can also improve water quality by reducing soil erosion and nutrient runoff. Soybean is a relatively low-tillage crop, which can help to reduce soil erosion and improve soil structure [181]. Furthermore, soybean residues left in the soil after harvest can help to reduce nutrient runoff by absorbing excess nutrients, such as nitrogen and phosphorus, from the soil. This can help to reduce nutrient pollution in waterways and improve water quality. In addition to reducing greenhouse gas emissions and improving water quality, soybean cultivation can also promote biodiversity by providing habitat for a variety of wildlife species.

## 5 Conclusions

By growing global population, the request for agricultural crops, including oilseed crops like soybean, will continue to increase in order to meet the demand for food and animal fodder. Iran is one of soybean production area in the world which faces significant tensions for soybean production due to biological and economic factors which affect sustainable agriculture negatively as well. Despite having the critical issues, the promotion usage of soybean in sustainable agricultural practices in future can enhance other crop productivity, reduce the environmental impacts, and boost long-term sustainability in Iran's agricultural sector. To achieve this advancement, this research suggested that 1) one leading approach is to implement the effective breeding programs to create new soybean varieties/inbred lines that are tolerant to different types of environmental stresses, including abiotic and biotic stresses, 2) the other method can be considered as the combination of molecular and classical breeding methods to develop improved soybean cultivars/genotypes, 3) the proper field management practices, including utilization of refined technological packages and timely application of inputs such as chemical or bio-fertilizers, herbicides, and pesticides based on expert recommendations should also be considered, 4) re-tooling and machine development can be effective way for seed multiplication projects, followed by their transfer to farmers to promote soybean production sustainability. Finally, 5) driving the policy makers, non-governmental organizations, and public and private breeders in the same direction can be profitable to promote soybean production based on consumer preferences, following by achieving successful soybean conservation agriculture production systems in the country. In fact, the successful future prospects of this precious significant crop in Iran will lead to diminish soybean imports based on domestic needs, economic growth by saving billions of dollars spending on its imports accompanying by growing food security.

## Ethics approval and consent to participate

Not applicable.

## Funding

No grant was available for this project.

## Consent for publication

Not applicable.

## Data availability statement

All data are within the manuscript.

## CRediT authorship contribution statement

**Parastoo Majidian:** Writing – original draft, Conceptualization. **Hamid Reza Ghorbani:** Writing – review & editing. **Mostafa Farajpour:** Writing – review & editing.

## Declaration of competing interest

The authors declare that they have no known competing financial interests or personal relationships that could have appeared to influence the work reported in this paper.
